# Bisalbuminémie: à propos de trois cas colligés au Laboratoire de Biochimie du Centre Hospitalier Universitaire Mohammed VI d’Oujda, Maroc, et revue de la littérature

**DOI:** 10.11604/pamj.2025.52.125.45856

**Published:** 2025-11-25

**Authors:** Fatima-Zahra Joudar, Dounia El Moujtahide, Elhoucine Sebbar, Mohammed Choukri

**Affiliations:** 1 Laboratoire de Biochimie, Centre Hospitalier Universitaire Mohammed VI d'Oujda, Oujda, Maroc,; 2 Faculté de Médecine et de Pharmacie d'Oujda, Université Mohammed Premier Oujda, Oujda, Maroc

**Keywords:** Bisalbuminémie, électrophorèse des protéines sériques, syndrome néphrotique, bêtalactamines, cas clinique, Bisalbuminemia, serum protein electrophoresis, nephrotic syndrome, beta-lactam antibiotics, case report

## Abstract

La bisalbuminémie est une anomalie qualitative rare et correspond à un épaulement ou à un dédoublement de la fraction de l'albumine sur le tracé électrophorétique, traduisant la présence chez un même individu d'une albumine plasmatique normale et d'une albumine de structure modifiée. Elle peut être héréditaire ou acquise et transitoire, dont les mécanismes physiopathologiques demeurent encore mal compris. Cette étude présente trois cas de bisalbuminémie observés au laboratoire de biochimie du Centre Hospitalier Universitaire Mohammed VI d'Oujda. Les résultats des électrophorèses montrent que toutes les bisalbuminémies rapportées étaient acquises et transitoires. Trois causes majeures de pseudo-bisalbuminémies ont été citées: l'administration des bêtalactamines, le syndrome néphrotique et la poussée de pancréatite chronique. En parallèle, à la lumière d'autres cas, nous rappelons les principales causes, génétiques ou acquises.

## Abstract (en)

Bisalbuminemia is a rare qualitative anomaly characterized by the occurrence of two distinct albumin bands or a single widened albumin band on serum protein electrophoresis, reflecting the presence of a normal albumin and a modified albumin in the same individual. It can be hereditary or acquired and transient, with physiopathological mechanisms that remain poorly understood. This study presents three cases of bisalbuminemia observed at the Biochemistry Laboratory of the Mohammed VI University Hospital in Oujda. Electrophoresis results showed that all reported cases were acquired and transient. Three major causes of pseudo-bisalbuminemia were identified: administration of beta-lactam antibiotics, nephrotic syndrome, and exacerbations of chronic pancreatitis. Additionally, in light of other reported cases, we here discuss the main genetic and acquired causes of this condition.

## Introduction

**Figure 1:**
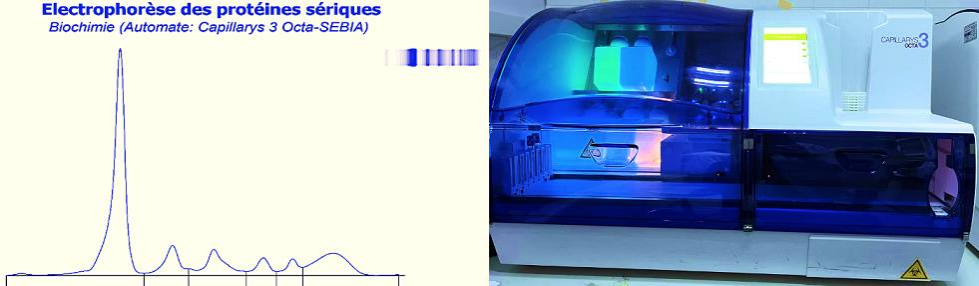
électrophorèses capillaires des protéines sériques normale obtenu à partir du CAPILLARYS 3 OCTA (SEBIA) et système d'électrophorèse CAPILLARYS 3 OCTA

L'électrophorèse des protéines sériques est un examen biologique couramment prescrit pour la détection d'anomalies qualitatives et quantitatives des protéines plasmatiques. Cette technique permet de séparer les protéines sériques selon leur mobilité électrophorétique en six fractions principales: l'albumine, les alpha-1 globulines (α1), les alpha-2 globulines (α2), les bêta-1 globulines (β1), les bêta-2 globulines (β2) et les gammaglobulines (γ), dans des conditions strictement standardisées de pH, de force ionique, de durée et d'intensité du courant appliqué (Figure 1).

Les bis-albuminémies sont définies par un dédoublement ou un épaulement de la fraction de l'albumine au niveau du tracé électrophorétique, lié à la coexistence chez un même individu de deux types d'albumine sérique, sans augmentation de leur concentration. Elles constituent un type rare de dys-albuminémie [1]. Il s'agit d'une anomalie protéique rare, congénitale ou acquise, qui est permanente dans la première éventualité et transitoire dans la seconde et à laquelle plusieurs causes sont actuellement reconnues.

Parmi les anomalies électrophorétiques rares, la bisalbuminémie se caractérise par un dédoublement ou un épaulement du pic d'albumine sur le tracé électrophorétique, traduisant la coexistence chez un même individu de deux formes distinctes d'albumine sérique, une normale et une modifiée sans élévation de la concentration totale d'albumine. Elle représente une dysalbuminémie peu fréquente, pouvant être héréditaire et permanente ou acquise et transitoire, selon son origine. Les formes acquises résultent généralement de modifications structurales ou fonctionnelles de la molécule d'albumine, induites par des pathologies ou des traitements médicamenteux.

Nous rapportons ici trois cas de bisalbuminémie acquise et transitoire, découverts fortuitement lors d'électrophorèses sériques réalisées au laboratoire de biochimie du CHU Mohammed VI d'Oujda. L'objectif de cette étude est de familiariser les cliniciens et les biologistes avec cette anomalie protéique rare, d'en discuter les mécanismes physiopathologiques possibles et d'en souligner les implications diagnostiques et pratiques. La bisalbuminémie demeure une anomalie très inconstante et peu documentée dans la littérature. Certaines formes restent encore sans étiologie clairement identifiée, malgré les progrès récents en biologie moléculaire et en électrophorèse capillaire. Notre étude contribue à enrichir le profil clinique et biologique de cette entité, en mettant en évidence des associations potentielles avec certaines affections acquises telles que le syndrome néphrotique, la pancréatite chronique et la prise de bêtalactamines. Ces observations permettent d'élargir la compréhension des mécanismes, du diagnostic et des implications cliniques de la bisalbuminémie, tout en ouvrant de nouvelles perspectives pour la recherche future sur les anomalies protéiques sériques.

## Patient et observation

### Cas n°1: syndrome néphrotique et bisalbuminémie acquise (Figure 2)

**Figure 2:**
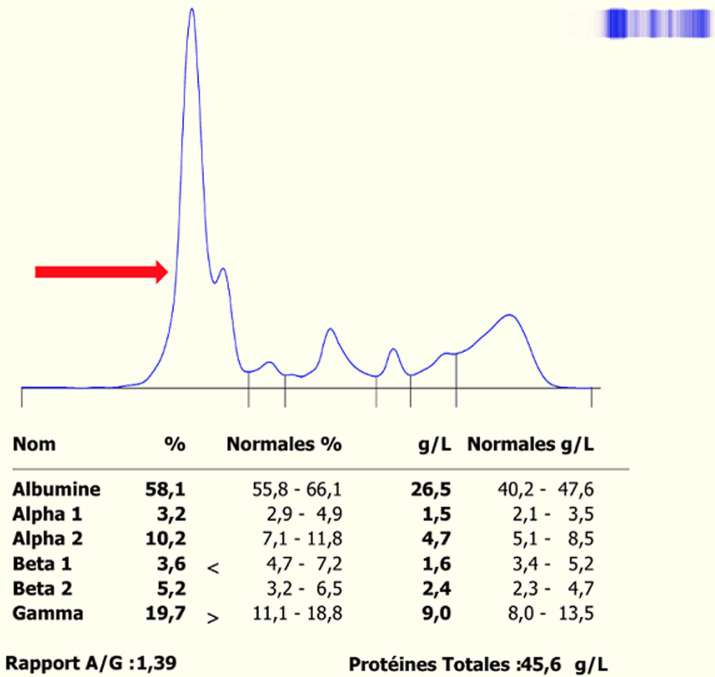
électrophorèse des protéines sériques du cas n°1 sur CAPILLARYS 3 OCTA-SEBIA

**Informations sur le patient:** enfant de 13 ans, issu d'un mariage non consanguin, sans antécédents pathologiques notables. Présente des œdèmes d'installation rapide aux membres inférieurs, associés à une asthénie.

**Contexte clinique et résultats d'examens:** l'échographie abdominopelvienne montre une hépatomégalie homogène sans lésion focale et des reins échogènes. Bilan biologique: urée et créatinine normales, hypoprotidémie à 45 g/L, hypoalbuminémie et hypo-α1-globulinémie, CRP légèrement augmentée (13 mg/L), hyperlipidémie (2,3 g/L, norme < 1,6 g/L) et hypertriglycéridémie (2,5 g/L, norme < 1,5 g/L), ECBU: hématurie microscopique, culture stérile.

**Évaluation diagnostique:** l'électrophorèse capillaire des protéines sériques (CAPILLARYS 3 OCTA, Sebia) révèle une bisalbuminémie bifide en “bonnet d'âne” d'amplitude inégale. Le profil électrophorétique évoque un syndrome néphrotique impur, confirmé par une protéinurie de 24 h à 4,3 g/24 h.

**Intervention thérapeutique et évolution:** le patient a été traité pour syndrome néphrotique selon le protocole hospitalier. L'enquête familiale a exclu une forme héréditaire. Après traitement, le tracé électrophorétique s'est normalisé, confirmant le caractère transitoire et acquis de la bisalbuminémie (Tableau 1).

**Tableau 1:** chronologie du cas n°1

Date / Délai	Événement clinique ou biologique	Observation / Résultat	Décision ou action médicale
J0	Apparition des œdèmes et asthénie	Suspicion de syndrome néphrotique	Prescription d'un bilan biologique complet
J+1	Électrophorèse des protéines sériques (CAPILLARYS 3 OCTA)	Aspect bifide de l'albumine → bisalbuminémie suspectée	Confirmation par dosage de la protéinurie
J+3	Bilan biologique complémentaire	Protéinurie à 4,3 g/24 h, hypoalbuminémie, hyperlipidémie	Diagnostic de syndrome néphrotique impur
J+5 à J+10	Mise en place du traitement spécifique	Amélioration clinique et biologique	Suivi hebdomadaire
J+30	Nouvelle électrophorèse	Normalisation du profil protéique	Confirmation d'une bisalbuminémie acquise et transitoire

**Consentement éclairé de la patiente:** le consentement éclairé a été obtenu de la part du parent du patient pour la publication de ses données cliniques et d'images dans ce manuscrit, conformément aux principes éthiques et à la réglementation en vigueur.

### Cas n°2: pancréatite chronique compliquée de bisalbuminémie

**Informations sur le patient:** patiente âgée de 77 ans, suivie pour pancréatite chronique. Admise pour prise en charge d'une poussée aiguë.

**Contexte clinique et résultats d'examens:** présente une douleur épigastrique en barre irradiant vers le dos. Lipasémie > 3 fois la limite supérieure. TDM abdominale: grande collection nécrosante remplaçant la majorité du tissu pancréatique. Aucune antibiothérapie instaurée.

**Figure 3:**
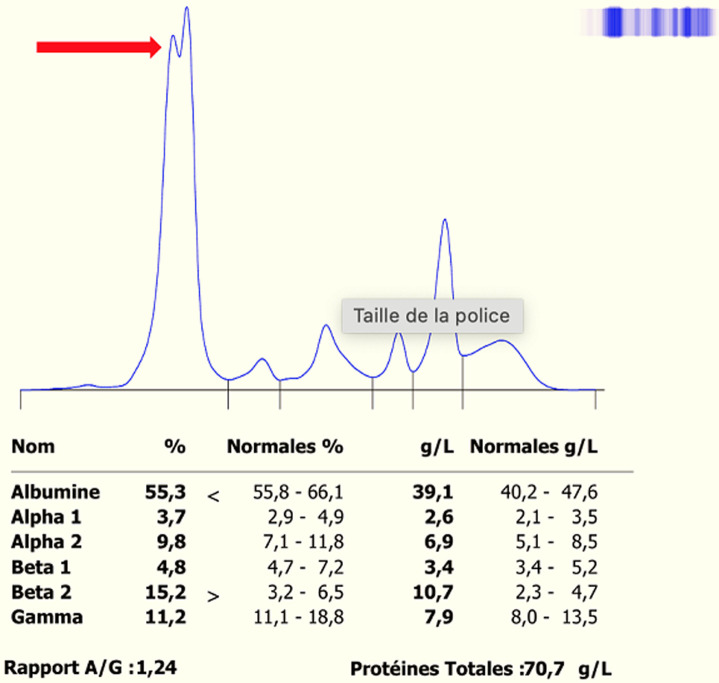
électrophorèse des protéines sériques du cas n°2 sur CAPILLARYS 3 OCTA-SEBIA

**Évaluation diagnostique:** l'électrophorèse capillaire des protéines sériques (Figure 3) montre un épaulement en amont du pic d'albumine, avec un taux inférieur à l'albumine normale. La bisalbuminémie est absente sur un profil d'électrophorèse réalisé un an auparavant, écartant la forme héréditaire.

**Intervention thérapeutique et évolution:** la prise en charge a consisté en traitement symptomatique et nutritionnel de la pancréatite. La bisalbuminémie est considérée acquise et transitoire, secondaire à une protéolyse partielle de l'albumine par les enzymes pancréatiques (Tableau 2).

**Tableau 2:** chronologie du cas n°2

Date / Délai	Événement clinique ou biologique	Observation / Résultat	Décision ou action médicale
J0	Admission pour douleur épigastrique en barre	Suspecte poussée de pancréatite aiguë	Bilan biologique et imagerie
J+1	TDM abdominopelvienne	Collection nécrosante pancréatique	Traitement symptomatique et nutritionnel
J+2	Lipasémie > 3N	Pancréatite confirmée	Surveillance rapprochée
J+3	Électrophorèse des protéines sériques	Apparition d'un épaulement sur le pic d'albumine → bisalbuminémie acquise	Observation sans intervention spécifique
J+30	Suivi biologique	Normalisation du profil électrophorétique	Disparition du double pic d'albumine

**Consentement éclairé de la patiente:** le consentement éclairé a été obtenu de la part du patient pour la publication de ses données cliniques et d'images dans ce manuscrit, conformément aux principes éthiques et à la réglementation en vigueur.

### Cas n°3: bisalbuminémie induite par bêtalactamines (**Figure 4**)

**Figure 4:**
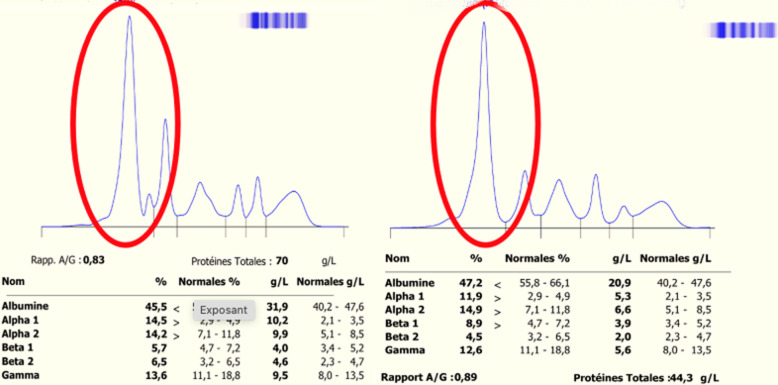
électrophorèse des protéines sériques du cas n°3 sur CAPILLARYS 3 OCTA-SEBIA, avant et après deux semaines d'arrêt de bêtalactamines

**Informations sur le patient:** patiente de 27 ans, suivie pour maladie de Crohn depuis 5 ans, avec des poussées inflammatoires récurrentes.

**Contexte clinique et résultats d'examens:** elle a été admise pour douleurs abdominales diffuses, fièvre persistante à 39,8°C et diarrhée liquide depuis 48h. Suspicion de surinfection dans un contexte de maladie inflammatoire chronique de l'intestin. Présente fatigue marquée et signes de déshydratation.

**Intervention thérapeutique:** interruption temporaire de la biothérapie par infliximab. Antibiothérapie à fortes doses de bêtalactamines instaurée pour traiter la surinfection (Tableau 3).

**Tableau 3:** chronologie du cas n°3

Date / Délai	Événement clinique ou biologique	Observation / Résultat	Décision ou action médicale
J0	Admission pour fièvre et douleurs abdominales dans un contexte de maladie de Crohn	Suspicion de surinfection bactérienne	Arrêt temporaire de l'infliximab
J+1	Mise sous antibiothérapie à forte dose (bêtalactamines)	Début du traitement	Surveillance biologique
J+5	Électrophorèse des protéines sériques	Dédoublement du pic d'albumine → bisalbuminémie suspectée	Poursuite du suivi
J+10	Persistance du double pic	Effet iatrogène suspecté	Décision d'arrêt des bêtalactamines
J+14	Nouvelle électrophorèse	Disparition du pic bifide	Confirmation de la bisalbuminémie transitoire et médicamenteuse

**Évaluation diagnostique:** l'électrophorèse des protéines sériques révèle un dédoublement du pic d'albumine, évoquant une bisalbuminémie acquise. Le caractère médicamenteux est retenu après exclusion d'autres causes.

**Suivi et évolution:** disparition complète du double pic deux semaines après l'arrêt des bêtalactamines, confirmant le caractère transitoire et iatrogène de l'anomalie.

**Consentement éclairé de la patiente:** le consentement éclairé a été obtenu de la part du patient pour la publication de ses données cliniques et d'images dans ce manuscrit, conformément aux principes éthiques et à la réglementation en vigueur.

## Discussion

Les résultats des électrophorèses des protéines sériques ont été analysés pour identifier la présence de bisalbuminémies. Un examen minutieux des antécédents cliniques des patients a permis de déterminer les causes possibles de cette anomalie. Le laboratoire de biochimie du CHU Med VI à Oujda utilise la technique d'électrophorèse capillaire (CAPILLARYS 3 OCTA® de la société SEBIA) (Figure 1), qui permet une séparation rapide et une automatisation complète de l'analyse. Le CAPILLARYS 3 OCTA de Sébia emploie l'électrophorèse capillaire pour l'analyse des protéines sériques. Cette méthode commence par l'injection des échantillons dans les capillaires via une aspiration à l'anode. La séparation des protéines s'effectue sous l'application d'une différence de potentiel de plusieurs milliers de volts entre les bornes du capillaire, favorisant la migration des protéines en fonction de leur charge et taille. La détection des protéines a lieu à 200 nm du côté cathodique, généralement par spectrophotométrie. Après un lavage des capillaires avec une solution de lavage et un tampon à pH basique, plusieurs fractions protéiques sont générées, qui sont ensuite analysées et quantifiées à l'aide d'un logiciel spécialisé et qui réalise à son tour une reconstitution qualitative du protéinogramme. On obtient six fractions des protéines suivant cet ordre: γ-globulines, β2 et β1-globulines, α2 et α1-globulines et albumine [2]. La détection de la bisalbuminémie est de plus en plus courante dans les laboratoires de biologie médicale, grâce aux avancées de l'électrophorèse capillaire. Cette technique, plus sensible, offre une résolution supérieure par rapport à l'électrophorèse traditionnelle sur gel d'agarose, et permet aujourd'hui de s'affranchir des limites de cette dernière.

Décrite pour la première fois par Scheurlen en 1955, la bisalbuminémie est une anomalie électrophorétique de l'albumine, sans caractère pathologique particulier, qui n'est souvent pas associée à des symptômes cliniques graves, bien que certains variants puissent influencer la liaison aux hormones comme la thyroxine ou aux stéroïdes, avec un potentiel d'interférence lors des tests de laboratoire ou des traitements hormonaux [3]. Elle se manifeste par un dédoublement de la fraction d'albumine sur le tracé d'électrophorèse des protéines sériques, et est caractérisée par la coexistence de deux types d'albumine sérique chez un même individu. Elles peuvent se présenter sous divers aspects, on distingue: un profil dit « en bonnet d'âne » avec deux pics distincts (qui peuvent avoir des hauteurs équivalentes ou non), un élargissement du pic, ou encore un épaulement en amont du pic d'albumine [4]. Ce type rare de dysalbuminémie peut être héréditaire et persistant, ou acquis et transitoire [1,3]. Sa prévalence est estimée entre 0,003% et 0,1%.

La bisalbuminémie héréditaire est permanente et de transmission autosomique (chromosome 4) codominante retrouvée chez plusieurs membres d'une même famille [5]. Elle résulte d'une mutation sur l'un des deux allèles au locus 4q13.3, chacun contribuant à environ la moitié de la production totale d'albumine, typiquement en état hétérozygote [3]. L'albumine modifiée peut migrer vers l'anode (charge positive) ou le cathode (charge négative) lors de l'électrophorèse, selon les caractéristiques de son point isoélectrique, c'est-à-dire le pH où elle n'a pas de charge nette. À un pH plus élevé que le point isoélectrique, elle devient négative et migre vers l'anode; à un pH plus bas, elle est positive et se dirige vers le cathode. Ces variations ne sont généralement pas associées à des conséquences pathologiques notables, bien qu'elles modifient la mobilité électrophorétique de la protéine [6]. Dans la majorité des cas observés, les variants d'albumine dans la bisalbuminémie migrent généralement de manière lente en électrophorèse, bien que certains variants puissent migrer plus rapidement. Ces variants présentent un rapport entre l'albumine normale et modifiée voisin de 1, en raison de légères modifications de charge nette, sans effets pathologiques significatifs [7]. La bisalbuminémie héréditaire est généralement sans symptôme clinique. Cependant, les variants d'albumine peuvent présenter une affinité modifiée pour certains ligands, tels que des médicaments ou des hormones, ce qui peut influencer le volume de distribution de ces substances et, dans de rares cas, engendrer des endocrinopathies. Parmi celles-ci, l'hyperthyroxinémie dysalbuminémique familiale se distingue par une affinité accrue de l'albumine pour la thyroxine libre (T4L), augmentant la fraction libre de cette hormone et donc modifiant les niveaux sériques des hormones thyroïdiennes (T4 libre (fT4) et, dans une moindre mesure, T3 libre (fT3)), ce qui peut conduire à des diagnostics erronés et à des traitements inappropriés, bien que leur fonction thyroïdienne soit en réalité normale [8]. Certains variants de l'albumine peuvent présenter une affinité modifiée par rapport à l'albumine normale pour des ions métalliques, des acides gras ou des médicaments, entraînant une élévation de leur concentration sanguine [9]. Le diagnostic de bisalbuminémie génétique est suspecté, puis posé pour un aspect électrophorétique superposable à des tracés postérieurs (donc le caractère permanent) et est fréquemment retrouvé chez plusieurs membres d'une même famille.

Dans un certain nombre de cas, la bisalbuminémie est transitoire et secondaire à plusieurs causes qui sont actuellement reconnues. Les bisalbuminémies secondaires ou pseudo-bisalbuminémie, surviennent généralement en raison de modifications structurales affectant une fraction de l'albumine circulante, dues à des ajouts ou pertes de certains constituants moléculaires [1]. Les bisalbuminémies acquises transitoires ont aussi été rapportées dans diverses pathologies, notamment le syndrome néphrotique (SN). C'est le cas de notre première observation (cas n°1) qui se distingue par la survenue chez un enfant de treize ans. Elle s'aligne avec la définition biochimique du syndrome néphrotique, caractérisé par une protéinurie supérieure à 3 g/24 h chez l'adulte (ou > 50 mg/kg/j chez l'enfant), une hypoalbuminémie inférieure à 30 g/L, et souvent une hyperlipidémie marquée par une élévation du cholestérol et/ou des triglycérides, cette dernière est due à l'augmentation de la synthèse hépatique des lipoprotéines en réponse à l'hypoalbuminémie; ces lipoprotéines et les chylomicrons migrent sur l'électrophorèse des protéines dans la zone de l'albumine, entraînant, en cas de dyslipidémie marquée, un épaississement artéfactuel, voire la formation d'une proéminence à la base du pic de l'albumine ou des α1-globulines [10]. Les dyslipidémies correspondant aux cas de molécules interférentes migrant dans la même zone que l'albumine sont les plus fréquemment rencontrées en électrophorèse capillaire. Le syndrome néphrotique est une cause inhabituelle de bisalbuminémie rarement rapportée dans la littérature [1].

La bisalbuminémie peut être rencontrée au cours d'une pancréatopathie comme celle que nous rapportons dans notre observation du cas n°2. Ceci s'explique par les enzymes pancréatiques, chymotrypsine et carboxypeptidases, activées par la trypsine qui sont à l'origine d'une protéolyse partielle de l'albumine, cette dernière amputée dans sa séquence primaire d'acides aminés présente une migration modifiée à l'électrophorèse. Les fractions digérées et non digérées de l'albumine migrent différemment lors de l'électrophorèse, formant ainsi deux pics distincts réalisant un aspect de bisalbuminémie sur le tracé électrophorétique [1]. C'est le cas de notre patiente (cas n°2) chez qui le diagnostic de pancréatite aigüe a été retenu. L'association entre pancréatite aigüe et bisalbuminémie est un cas rare, mais qui a été déjà rapporté par certains auteurs dans la littérature.

La bisalbuminémie médicamenteuse représente une forme particulière de bisalbuminémie acquise, et se rencontre lors de l'administration d'antibiotiques, tout particulièrement pour des posologies prolongées et élevées en bêtalactamines. La survenue de bisalbuminémie est interprétée comme signe de surdosage, et elle se voit particulièrement en cas d'insuffisance rénale. Le cycle bêta-lactame se fixe sur le groupe aminé de lysine de l'albumine par son groupement carbamyle, entraînant une modification de la charge de la protéine et donc de sa migration, cette anomalie est détectée de manière fortuite lors d'examens de routine. Chez un individu sain recevant une faible dose de bêtalactamines, les récepteurs de l'albumine ne sont pas complètement saturés; en revanche, en cas d'hypoalbuminémie ou de posologie élevée, les sites de liaison se saturent rapidement, entraînant une augmentation de la fraction libre, active ou potentiellement toxique, de l'antibiotique, ainsi l'électrophorèse des protides peut être considérée comme un moyen de surveillance d'un surdosage par les bêtalactamines. Le pic apparaît généralement entre le troisième et le huitième jour de traitement et est plus marqué pour les doses plus élevées d'antibiotiques. Dans la littérature, l'association entre la bisalbuminémie et le traitement par antibiotiques bêtalactamines est déjà bien documentée.

La littérature rapporte des études qui ont montré que la prévalence de la bisalbuminémie est également élevée chez les patients atteints de maladies rénales auto-immunes telles que le lupus érythémateux disséminé et la glomérulonéphrite, et aussi dans certaines pathologies telles que le myélome multiple à IgM et à IgA. D'autres facteurs de risque ont été décrits incluant le tabagisme, l'alcoolisme et l'obésité. Des études ont également montré que la bisalbuminémie est plus fréquente chez les personnes atteintes de certaines maladies génétiques telles que la maladie de Fabry ou la maladie d'Alport. Les métastases hépatiques d'adénocarcinomes d'origine digestive peuvent également induire une bisalbuminémie.

## Conclusion

Tous les cas de bisalbuminémie recensés dans notre travail sont de nature transitoire et acquise. Bien que rare, elle peut être observée dans diverses conditions cliniques, notamment le syndrome néphrotique et l'utilisation de certains antibiotiques comme les bêtalactamines, et les pancréatopathies. Étant donné que de nombreux laboratoires de biologie clinique ont désormais remplacé la technique classique de l'électrophorèse sur gel d'agarose par l'électrophorèse capillaire (comme le CAPILLARYS de la société Sebia), la fréquence de détection de la bisalbuminémie devrait augmenter parallèlement à l'utilisation de l'électrophorèse capillaire. Ce travail avait pour objectif principal de mieux comprendre la bisalbuminémie, en particulier dans le contexte médicamenteux, et de mettre en lumière les mécanismes sous-jacents à cette anomalie afin de familiariser les cliniciens, le personnel de laboratoire et les scientifiques avec cette dernière. À travers l'analyse des cas cliniques, nous avons pu mettre en évidence les liens entre cette anomalie protéique et des traitements spécifiques, comme les bêtalactamines, ainsi que son association avec des pathologies telles que le syndrome néphrotique. L'étude des différents cas nous a permis d'approfondir la compréhension de cette condition encore mal connue et de souligner l'importance de la surveillance clinique dans les cas traités par certains médicaments, afin de prévenir toute confusion diagnostique et adapter au mieux la prise en charge.

## References

[1] Rar L, Uwingabiye J, Biaz A, El Mechtani S, Dami A, Ouzzif Z (2018). Association insolite d'une cholestase ictérique à une bis-albuminémie acquise. Revue Francophone des Laboratoires.

[2] Sebia CAPILLARYS PROTEIN(E) 6 Rxonly.

[3] Minchiotti L, Caridi G, Campagnoli M, Lugani F, Galliano M, Kragh-Hansen U (2019). Diagnosis, Phenotype, and Molecular Genetics of Congenital Analbuminemia. Front Genet.

[4] Lefrère B, Dedôme E, Garcia-Hejl C, Ragot C, Chianea D, Delacour H (2018). Les bisalbuminémies: à propos d'un cas [Bisalbuminemia: A case report]. Rev Med Interne.

[5] Yousra E, Kawtar L, Sara C, Asmaa M, Mohammed BG, Nabiha K (2023). Etiologies et prévalence des bisalbuminémies associées aux néphropathies découvertes sur électrophorèse des protéines sériques sur capillaire au Laboratoire de Biochimie CHU Ibn Rochd de Casablanca Maroc. International Journal of Innovation and Applied Studies.

[6] Garfin DE (1990). [35] Isoelectric focusing. In Methods in enzymology.

[7] Madison J, Galliano M, Watkins S, Minchiotti L, Porta F, Rossi A (1994). Genetic variants of human serum albumin in Italy: point mutants and a carboxyl-terminal variant. Proc Natl Acad Sci U S A.

[8] Dieu X, Bouzamondo N, Briet C, Illouz F, Moal V, Boux de Casson F (2020). Familial Dysalbuminemic Hyperthyroxinemia: An Underdiagnosed Entity. J Clin Med.

[9] El Boukhrissi F, Balouch L, Moudden K, Baaj M, Hommadi A, Bamou Y (2016). Bisalbuminémie survenant en dehors des situations habituelles [Bisalbuminemia occurring outside usual situations]. Rev Med Interne.

[10] Galezowski N, Jouanique-Bayrod C, Dazza F, Gehrig D, Trivin F, Herreman G (1997). Bisalbuminémie révélant une hyperparathyroïdie primaire avec faux kyste du pancréas fistulisé. La Revue de médecine interne.

